# Noncardiac anomalies in children with congenital heart disease

**DOI:** 10.3389/fcvm.2023.1293210

**Published:** 2023-11-20

**Authors:** Xianghui Huang, Yuan Gao, Weicheng Chen, Wei Sheng, Guoying Huang

**Affiliations:** ^1^ Cardiovascular Center, Children’s Hospital of Fudan University, Shanghai, China; ^2^ Fujian Key Laboratory of Neonatal Diseases, Xiamen Children’s Hospital, Fujian, China; ^3^ Shanghai Key Laboratory of Birth Defects, Shanghai, China; ^4^ Unit of Early Intervention of Genetically Related Childhood Cardiovascular Diseases, Shanghai, China

**Keywords:** congenital heart defects, noncardiac anomalies, Chinese children, retrospective analyses, phenotype

## Abstract

**Introduction:**

Noncardiac anomalies (NCAs) in patients with congenital heart defects (CHDs) are crucial for perioperative management and etiology studies. This study aimed to investigate NCAs in Chinese children with CHDs.

**Methods:**

Medical records for CHD-diagnosed children hospitalized from 1 January 2015 to 31 December 2019 were collected and subjected to retrospective analyses to excavate potential association rules between CHDs and noncardiac malformations.

**Results:**

A total of 3,788 CHD patients were included in this study. The main phenotypes of CHD were Ventricular Septal Defect (VSD, 33.69%), Atrial Septal Defect (ASD, 12.72%), and Tetralogy of Fallot (TOF, 5.54%). A total of 887 (23.42%) cases showed noncardiac anomalies, which were mainly associated with the central nervous system (34.61%), nose/ear/mandibular/face (19.39%), genitourinary system (15.78%), and musculoskeletal system (15.56%). Compared to other CHD subtypes, septal defects had a lower percentage of associated NCAs (*P *= 3.7 × 10^−9^) while AVSD had a higher percentage (*P *= 0.0018).

**Disscussion:**

NCAs are prevalent among CHD-diagnosed children in China, and the spectrums of NCAs in different CHD subcategories were different.

## 1. Introduction

Congenital heart defects (CHDs) are a leading cause of infant mortality and the most commonly diagnosed major congenital anomaly worldwide (1, 2) with a prevalence of 8.98 per 1,000 live births in China (3). Although diagnostic capabilities and surgical strategies have significantly advanced, CHD remains a costly and onerous disease burden in many countries (4, 5). CHDs manifest as diverse structural abnormalities of the heart and great vessels that arise during embryonic development. The complexity of structural anomalies, defect severity, and the presence of comorbidities are essential determinants of CHD outcomes (6).

It is important to identify whether a CHD presents as an isolated condition or has developed in combination with noncardiac anomalies, as is frequently the case. Multiple organs originate from a common germ layer and can be regulated by shared signaling pathways (7). Moreover, chromosomal aberrations, which represent a leading cause of CHDs, alter the activities of multiple genes that may have different functions in embryogenesis. CHD comorbidities affecting other systems have a significant impact on clinical course. Despite advances in cardiac surgery and perioperative management, Eskedal et al. (8) reported that survival has not improved for CHD-diagnosed children with extracardiac anomalies. A 15-year follow-up study showed that noncardiac anomalies (NCAs) had a more significant effect on mortality in patients with CHDs than potential heart disease (9). Indeed, some have recommended that prediction modeling of thoracic surgery mortality risk for CHD patients could be augmented by adding a covariate that represents noncardiac congenital anatomic abnormalities (10). NCAs can increase the risk of postoperative complications, such as respiratory complications with heterotaxy (11), and children with CHD undergoing noncardiac surgery can have an increased mortality risk (12). Consequently, to identify optimal perioperative and long-term management of patients with CHDs and to elucidate the etiology of congenital defects, a detailed description of coexisting NCAs in children with CHD is necessary.

Reported frequencies of NCAs in CHD-diagnosed patients range widely from 4.53% to 50% (13–15). Different types of CHD are associated with distinct incidences of extracardiac comorbidities. For example, the NCA risk has been reported to be elevated among CHD patients with a cardiac looping defect, conotunal defect (CTD), or atrial septal defect (ASD) and reduced among CHD patients with a ventricular septal defect (VSD) or Ebstein anomaly (16, 17). Moreover, specific associations have been identified between common truncus and limb reduction defects and between great vessel-transposition and situs inversus (18). Piran found a trend toward a higher prevalence of major congenital extracardiac anomalies involving the musculoskeletal and genitourinary systems in adults with tetralogy of Fallot (TOF) (19). Thus far, there are limited data in the literature regarding the occurrence of NCAs in CHD-diagnosed children in China, and this study aimed to describe the proportions and distributions of NCAs in Chinese children of Han ethnicity who have been diagnosed with a CHD.

## 2. Materials and methods

### 2.1. Study population

Children diagnosed with a CHD and hospitalized in the Department of Cardiac Surgery, Children's Hospital of Fudan University from 1 January 2015 to 31 December 2019 were eligible for inclusion. Data collection and analysis processes were conducted with the permission of and under the guidance of the ethics committee. Written informed consent to participate was obtained from the guardians. Medical histories, surgical records, imaging data, outpatient medical records, and genetic test results of enrolled patients were collected and examined retrospectively. CHD diagnoses were confirmed by clinical assessment, echocardiography, cardiac catheterization, and, if necessary, surgical observations. Patent foramen ovale, patent ductus arteriosus (PDA) in premature infants, and a PDA that resolved within 3 months after birth were excluded. The analyses did not include patients with cardiomyopathies, cardiac arrhythmias, or primary pulmonary hypertension.

### 2.2. Phenotype classification

Individual patients' CHDs were classified based on anatomy and etiology (20–23) into the level 1 and 2 categories (Table 1), where the latter are subcategories of the former. Level 1 categories included the following: septal defects, atrioventricular canal defect (AVSD), conotruncal cardiac defect (CTD), left ventricular outflow tract obstruction (LVOTO), right ventricular outflow tract obstruction (RVOTO), abnormal cell growth, heterotaxy (HTX), others, and associations.

**Table 1:** Distribution of CHD subtypes and corresponding number of NCAs.

CHD phenotypes^a^	*n* (%)^b^	Sex ratio (M/F)	Number of noncardiac malformation, *n* (%)^c^			
			0	1	2	≥3
Septal defect	2,155 (56.89)	1.11	1,727 (80.14)	277 (12.85)	115 (5.34)	36 (1.67)
VSD	1,276 (33.69)	1.34	1,056 (82.76)	146 (11.44)	54 (4.23)	20 (1.57)
ASD	482 (12.72)	0.88	361 (74.90)	76 (15.77)	37 (7.68)	8 (1.66)
VSD + ASD	397 (10.48)	0.83	310 (78.09)	55 (13.85)	24 (6.05)	8 (2.02)
AVSD	124 (3.27)	0.8	80 (64.52)	22 (17.74)	17 (13.71)	5 (4.03)
AVSD	108 (2.85)	0.83	70 (64.81)	18 (16.67)	17 (15.74)	3 (2.78)
AVSD + OTO	16 (0.42)	0.60	10 (62.50)	4 (25.00)	0 (0.00)	2 (12.50)
CTD	524 (13.83)	1.94	400 (76.34)	80 (15.27)	37 (7.06)	7 (1.34)
PTA	9 (0.24)	0.50	6 (66.67)	2 (22.22)	0 (0.00)	1 (11.11)
DTGA-IVS	48 (1.27)	3.36	43 (89.58)	4 (8.33)	1 (2.08)	0 (0.00)
DTGA-IVS + OTO	11 (0.29)	*F* = 0	5 (45.45)	4 (36.36)	2 (18.18)	0 (0.00)
DTGA + VSD	65 (1.72)	2.61	56 (86.15)	8 (12.31)	1 (1.54)	0 (0.00)
TOF	210 (5.54)	1.76	163 (77.62)	28 (13.33)	14 (6.67)	5 (2.38)
PA-VSD	43 (1.14)	1.05	32 (74.42)	6 (13.95)	5 (11.63)	0 (0.00)
DORV	77 (2.03)	1.85	58 (75.32)	13 (16.88)	6 (7.79)	0 (0.00)
APW	7 (0.18)	1.33	3 (42.86)	2 (28.57)	2 (28.57)	0 (0.00)
IAA	26 (0.69)	1.89	19 (73.08)	5 (19.23)	2 (7.69)	0 (0.00)
Vasc ring malformations	28 (0.74)	3.00	15 (53.57)	8 (28.57)	4 (14.29)	1 (3.57)
LVOTO	66 (1.74)	1.64	46 (69.7)	16 (24.24)	1 (1.52)	3 (4.55)
HLH	3 (0.08)	2.00	2 (66.67)	1 (33.33)	0 (0.00)	0 (0.00)
Valvular/supravalvular aortic stenosis or other malformation of aorta or the aortic valve	63 (1.66)	1.63	44 (69.84)	15 (23.81)	1 (1.59)	3 (4.76)
RVOTO	242 (6.39)	1.00	192 (79.34)	37 (15.29)	11 (4.55)	2 (0.83)
Pulmonary artery/valve atresia or other malformations of pulmonary artery/valve	146 (3.85)	0.85	119 (81.51)	19 (13.01)	6 (4.11)	2 (1.37)
Eebstein	12 (0.32)	2.00	8 (66.67)	3 (25)	1 (8.33)	0 (0.00)
TA-VSD/TA + PA-IVS	6 (0.16)	1.00	3 (50.00)	2 (33.33)	1 (16.67)	0 (0.00)
DCRV	59 (1.56)	1.19	45 (76.27)	12 (20.34)	2 (3.39)	0 (0.00)
HRH	19 (0.50)	1.38	17 (89.47)	1 (5.26)	1 (5.26)	0 (0.00)
Abnormal cell growth	75 (1.98)	1.78	59 (78.67)	11 (14.67)	4 (5.33)	1 (1.33)
APVD	56 (1.48)	2.73	46 (82.14)	6 (10.71)	3 (5.36)	1 (1.79)
Cor triatriatum	19 (0.50)	0.58	13 (68.42)	5 (26.32)	1 (5.26)	0 (0.00)
Others	151 (3.99)	0.61	108 (71.52)	30 (19.87)	10 (6.62)	3 (1.99)
CCTGA	6 (0.16)	2.00	4 (66.67)	2 (33.33)	0 (0.00)	0 (0.00)
SV	7 (0.18)	2.50	6 (85.71)	1 (14.29)	0 (0.00)	0 (0.00)
Isolated malformations of great arteries	3 (0.08)	2.00	1 (33.33)	2 (66.67)	0 (0.00)	0 (0.00)
Isolated malformation of the coronary arteries	15 (0.40)	0.36	12 (80.00)	2 (13.33)	0 (0.00)	1 (6.67)
Isolated malformations of heart valve	38 (1.00)	0.46	28 (73.68)	7 (18.42)	3 (7.89)	0 (0.00)
Isolated PDA	82 (2.16)	0.58	57 (69.51)	16 (19.51)	7 (8.54)	2 (2.44)
HTX	87 (2.30)	1.35	8 (9.2)	62 (71.26)	13 (14.94)	4 (4.6)
HTX + no CVM	5 (0.13)	*F* = 0	4 (80.00)	0 (0.00)	0 (0.00)	1 (20.00)
HTX + simple CVM	37 (0.98)	0.76	2 (5.41)	26 (70.27)	7 (18.92)	2 (5.41)
HTX + complex CVM	45 (1.19)	1.81	2 (4.44)	36 (80.00)	6 (13.33)	1 (2.22)
Associations	364 (9.61)	1.38	281 (77.2)	52 (14.29)	20 (5.49)	11 (3.02)
AVSD + CTD	10 (0.26)	1.00	2 (20.00)	5 (50.00)	1 (10.00)	2 (20.00)
Septal defects + APVD	104 (2.75)	1.48	87 (83.65)	10 (9.62)	6 (5.77)	1 (0.96)
Septal defects + RVOTO	95 (2.51)	1.07	73 (76.84)	14 (14.74)	7 (7.37)	1 (1.05)
Septal defects + LVOTO	123 (3.25)	1.51	95 (77.24)	19 (15.45)	5 (4.07)	4 (3.25)
RVOTO + LVOTO	9 (0.24)	2.00	6 (66.67)	1 (11.11)	1 (11.11)	1 (11.11)
CTD + LVOTO	17 (0.45)	2.40	15 (88.24)	1 (5.88)	0 (0.00)	1 (5.88)
CTD + APVD	6 (0.16)	1.00	3 (50.00)	2 (33.33)	0 (0.00)	1 (16.67)
TOTAL	3,788 (100)	1.20	2,901 (76.58)	587 (15.50)	228 (6.02)	72 (1.90)

VSD, ventricular septal defect; ASD, atrial septa defect; AVSD, atrioventricular septal defect; OTO, outflow tract obstruction, including LVOTO (left ventricular outflow tract obstruction) and RVOTO (right ventricular outflow tract obstruction); CTD: conotruncal defect; PTA, persistent truncus arteriosus; D-TGA, dextro-transposition of the great arteries; IVS, intact ventricular septum; TOF, tetralogy of fallot; PA, pulmonary atresia; DORV, double outlet right ventricle; APW, aortopulmonary window; IAA, interruption of aortic arch, including type A/B/C; vasc ring malformations, including right arch and sling; HLH, hypoplastic left ventricle; TA, tricuspid atresia; DCRV, double-chambered right ventricle; HRH, hypoplastic right ventricle; APVD, anomalous pulmonary venous drainage; CCTGA, congenitally corrected transposition of the great arteries; SV, single ventricle; PDA, patent ductus arteriosus; HTX, heterotaxy or situs inversus totalis; CVM, refer to above structural cardiovascular malformations, and complex CVM refers to more than one above cardiovascular malformations.

^a^ CHD level 1 subcategories are represented in bold font centered on the center, and the level 2 subcategories included in each level 1 subcategories are aligned to the left below each other.

^b^ Percentage denominator was total *N* of 3,788.

^c^ Percentage denominator was total number of each CHD subcategories.

Diagnosed NCAs were summarized and aggregated according to affected systems. All developmental delays, including growth retardation, intellectual disability, mental delays, learning difficulties, and language/motor deficits, were classified as central nervous system (CNS) anomalies (24). We ignored syndrome diagnoses to focus on phenotypes. Syndrome-associated noncardiac phenotypes were disassociated and classified according to their respective affected systems. Anomalies within the same affected system were counted as one. CHD cases with and without NCAs are referred to as associated CHD cases and isolated CHD cases, respectively.

### 2.3. Statistical analysis

R (version 4.1.2), R Studio (version 1.4.1717), and SPSS (IBM version 20) were used for statistical analysis. Analysis of description was used to provide the whole picture of CHD and NCAs; Pearson's *χ*^2^ test or Fisher's exact test was applied to compare the proportions of isolated CHD between different genders and different CHD subtypes. The apriori principle (25) was used to analyze CHD diagnosis-to-NCA association, setting thresholds for support and confidence at 0.01 and 0.1, respectively. Rules with a lift ≥ 3 were considered of value.

## 3. Results

### 3.1. Proportions of congenital heart defects

A total of 3,788 CHD-diagnosed children were identified retrospectively, of which 2,065 (54.51%) were male and 1,723 were female (45.49%) with a median age of 3.96 years (IQR, 2.56–5.39). The proportions of level 1 and level 2 diagnostic classifications are reported in Table 1 with sex ratios and the number of co-occurring anomalies. The most common level 1 diagnoses were septal defect (56.89%), CTD (13.83%), and RVOTO (6.39%); besides different septal defects TOF (5.54%), pulmonary artery/valve atresia or other malformations of the pulmonary artery/valve (3.85%), DORV (2.03%), valvular/supravalvular aortic stenosis or other malformation of the aorta or the aortic valve (1.66%), and dextro-transposition of the great arteries (D-TGA, 1.27%) were common level 2 diagnoses. Furthermore, 3.25% children had both septal defects and LVOTO and 2.75% children had septal defects and RVOTO.

### 3.2. Noncardiac malformations in children with CHDs

A total of 887 (23.42%) cases of CHD were associated with noncardiac malformations, comprising 587 (15.50%) with one malformation, 228 (6.02%) with two malformations, and 72 (1.90%) with three or more malformations (Table 1).There were 1,284 noncardiac malformations among the 887 children, including 307 (34.61%) in the nervous system, 172 (19.39%) in the nose/ear/mandibular/face, and 140 (15.78%) in the musculoskeletal system (Table 2). The most common discrete NCA phenotype by far was developmental delay (*n* = 262, 29.54%), inguinal hernia (*n* = 83, 9.36%), and cryptorchidism and hydrocele (*n* = 77, 8.68%).

**Table 2:** Distribution of NCAs in 887 children with associated CHD.

Noncardiac anomaly	i (%)^a^
Central nervous system anomalies	307 (34.61)
Archinencephaly	69 (7.78)
Spina bifida	5 (0.56)
Hydrocephaly	9 (1.01)
Development delay, intellectual disability, mental delay, learning difficulties, language and motor deficit	262 (29.54)
Nose, ear, mandibular, and face anomalies	172 (19.39)
Genitourinary system anomalies	140 (15.78)
Renal or bladder dysplasia and hydronephrosis	20 (2.25)
Hypospadias and urethral valve	35 (3.95)
Abnormal genitalia	23 (2.59)
Cryptorchidism and hydrocele	77 (8.68)
Musculoskeletal anomalies	138 (15.56)
Polydactyly, syndactyly, and trigger finger	52 (5.86)
Developmental dysplasia of hip and other malformations of limbs	30 (3.38)
Spine anomalies	21 (2.37)
Skull deformity	4 (0.45)
Chest wall deformity and rib dysplasia	21 (2.37)
Torticollis and hypotonia	20 (2.25)
Heterotaxy	108 (12.18)
Abdominal wall deformity	98 (11.05)
Inguinal hernia	83 (9.36)
Umbilical hernia and omphalocele	15 (1.69)
Digestive system anomalies	56 (6.31)
Esophageal stricture/atresia and tracheoesopahgeal fistula	9 (1.01)
Biliary atresia and cholestasis	21 (2.37)
Hirschsprung's disease, stenosis/atresia of digestive tract, malrotation, and fistula	13 (1.47)
Annular pancreas and ectopic pancreas	5 (0.56)
Anal atresia and cloacal malformations	16 (1.80)
Respiratory system anomalies	55 (6.20)
Upper/lower airway stenosis or dysplasia	46 (5.19)
Pulmonary sequestration and other lung aplasia	9 (1.01)
Congenital hypothyroidism and other endocrine and metabolic disease	47 (5.30)
Hemangioma and lymphangioma	47 (5.30)
Cleft lip and/or palate	25 (2.82)
Absence of thymus and other immunodeficiency	19 (2.14)
Eye anomalies	19 (2.14)
Cataract, glaucoma and other abnormal structure of cornea, sclera, iris, and retina	13 (1.47)
Visual impairment	6 (0.68)
Hearing impairment	15 (1.69)
Miscellaneous/Others^b^	38 (4.28)
Total^c^	1,284

^a^ Percentage denominator was 887 patients with associated CHD.

^b^ Includes aplastic anemia, idiopathic thrombocytopenic purpura, thalassemia, vitiligo, alopecia areata, lipoma, skin growth, etc

^c^ “TOTAL” represents the sum of noncardiac anomalies in 887 children with associated CHD, and anomalies within the same affected system were counted as one.

### 3.3. NCA spectra for CHD subcategories

The level 1 diagnoses with the highest percentages of isolated CHDs were septal defects, wherein about four fifths of each were isolated (Table 1). Conversely, besides heterotaxy, the diagnosis with the greatest percentage of associated CHDs was AVSD, wherein almost two fifths of the patients have associated CHDs (Table 1). Statistical analysis proved that septal defects, especially VSD (*P *= 3.7 × 10^−9^) and D-TGA with an intact ventricular septum (D-TGA + IVS, *P *= 0.038), are less likely to associated with NCAs. Furthermore, there were a higher probability of combined NCAs in children with AVSD (*P *= 0.002) and vasc ring malformations (*P *= 0.007).

Although NCAs involving the CNS, nose/ear/mandibular/face, genitourinary system, and musculoskeletal system were common for most CHD subtypes, the distribution of NCAs differed slightly (Table 3). NCAs affecting the CNS were most frequently observed in AVSD and RVOTO cases, wherein approximately one third of patients were affected. Nose/ear/mandibular/face anomalies were observed in more than one fifth of AVSD cases. Children in groups of septal defects or groups of associations showed relatively larger proportions of combined musculoskeletal anomalies (12.16% and 12.00%) and abdominal wall deficiency (9.48% and 8.80%). Genitourinary malformations were less prevalent in LVOTO-diagnosed children whereas respiratory system NCAs and digestive system NCAs were apparently common in children with CTD and LVOTO, respectively. Laterality defects were notably common in heterotaxy cases and, albeit to a lesser extent, CTD cases (Table 3). Furthermore, taking gender into account, among children with septal defects, especially those with VSD, PA-VSD, pulmonary artery/valve atresia, or other malformation of the pulmonary artery/valve and double-chambered right ventricule (DCRV), males were more likely to have associated CHD than females (*P* < 0.05), whereas the situation is opposite in LVOTO and RVOTO diagnostic groups.

**Table 3:** Distribution of NCAs associated with each CHD subcategory^a^.

CHDs NCAs	Septal defect	AVSD	CTD	LVOTO	RVOTO	Abnormal cell growth	Others	HTX	Associations
Central nervous system anomalies	160 (25.28)	22 (30.56)	36 (20.57)	9 (31.03)	21 (31.82)	9 (40.91)	9 (15.25)	9 (8.74)	32 (25.60)
Nose, ear, mandibular, face anomaly	88 (13.90)	16 (22.22)	27 (15.43)	3 (10.34)	9 (13.64)	3 (13.64)	8 (13.56)	4 (3.88)	14 (11.20)
Genitourinary	72 (11.37)	7 (9.72)	21 (12.00)	2 (6.90)	10 (15.15)	4 (18.18)	10 (16.95)	1 (0.97)	13 (10.40)
Musculoskeletal	77 (12.16)	7 (9.72)	16 (9.14)	3 (10.34)	6 (9.09)	0 (0.00)	8 (13.56)	6 (5.83)	15 (12.00)
Laterality defects	8 (1.26)	3 (4.17)	12 (6.86)	0 (0.00)	3 (4.55)	0 (0.00)	3 (5.08)	72 (69.9)	7 (5.60)
Respiratory system	23 (3.63)	1 (1.39)	15 (8.57)	1 (3.45)	0 (0.00)	1 (4.55)	4 (6.78)	3 (2.91)	7 (5.60)
Digestive system	31 (4.90)	3 (4.17)	8 (4.57)	4 (13.79)	1 (1.52)	0 (0.00)	0 (0.00)	3 (2.91)	6 (4.80)
Abdominal wall	60 (9.48)	3 (4.17)	8 (4.57)	2 (6.90)	5 (7.58)	2 (9.09)	5 (8.47)	2 (1.94)	11 (8.80)
Endocrine and metabolic	25 (3.95)	3 (4.17)	4 (2.29)	3 (10.34)	3 (4.55)	0 (0.00)	3 (5.08)	0 (0.00)	6 (4.80)
Hemangioma, lymphangioma	25 (3.95)	1 (1.39)	10 (5.71)	1 (3.45)	1 (1.52)	2 (9.09)	2 (3.39)	0 (0.00)	5 (4.00)
Cleft lip and/or palate	18 (2.84)	1 (1.39)	0 (0.00)	0 (0.00)	2 (3.03)	0 (0.00)	0 (0.00)	2 (1.94)	2 (1.60)
Immunodeficiency	7 (1.11)	2 (2.78)	8 (4.57)	0 (0.00)	1 (1.52)	0 (0.00)	0 (0.00)	0 (0.00)	1 (0.80)
Eye	5 (0.79)	2 (2.78)	4 (2.29)	0 (0.00)	1 (1.52)	0 (0.00)	5 (8.47)	0 (0.00)	2 (1.60)
Hearing impairment	8 (1.26)	1 (1.39)	2 (1.14)	0 (0.00)	1 (1.52)	0 (0.00)	1 (1.69)	0 (0.00)	2 (1.60)
Others	26 (4.11)	0 (0.00)	4 (2.29)	1 (3.45)	2 (3.03)	1(4.55)	1(1.69)	1(0.97)	2(1.60)
Total: NCAs/associated CHDs^b^	633/428	70/44	175/124	29/20	66/50	22/16	59/43	103/79	125/83

^a^ Number of subjects with the certain CHD subcategories and NCAs are indicated; percentages in parentheses indicate the portion of specified NCA among all NCAs of children with each CHD phenotype in the header.

^b^ NCAs refers to the total number of NCAs within the population with specific CHD subcategories while associated CHDs refers to the total number of patients with combined NCAs.

### 3.4. Co-occurrence of CHDs and NCAs

With support and confidence levels set to 0.01 and 0.1, respectively, we attained 213 rules between CHD subtypes and NCAs. After filtering out low-value rules (lift < 3), 11 rules were retained (Table 4), showing the reliability or value of the rule which indicated the following potential CHD–NCA co-occurrence relationships: CCTGA-SV–laterality defect (lift = 13.02); CTD, RVOTO–laterality defect NCA (lift = 9.01); heterotaxy–laterality defect NCA (lift = 6.93); vasc ring malformations–respiratory system NCA (lift = 5.19); VSD, PDA, and CNS anomalies-LVOTO (lift = 3.66).

**Table 4:** Valuable rules with association analysis^a^.

Type^b^	lhs^c^	rhs^c^	Count	Support^d^	Confidence^d^	Lift^d^	Coverage^d^	Lift^d^	Conviction^d^	FishersExactTest^V^
NCA	CNS, Miscellaneous	Face	13	0.0156	0.6842	3.3370	0.0228	3.3370	2.5174	6.4871 × 10^−6^
NCA	Face, CNS	Miscellaneous	13	0.0156	0.1512	3.3176	0.1031	3.3176	1.1244	3.8934 × 10^−5^
CHD_NCA	laterality defect (*N*)^f^	CCTGA, SV	13	0.0156	0.2653	13.0156	0.0588	13.0156	1.3334	3.8326 × 10^−14^
CHD_NCA	CCTGA, SV	Laterality defect (*N*)	13	0.0156	0.7647	13.0156	0.0204	13.0156	4.0003	3.8326 × 10^−14^
CHD_NCA	CTD, RVOTO	Laterality defect (*N*)	9	0.0108	0.5294	9.0108	0.0204	9.0108	2.0001	6.7795 × 10^−8^
CHD_NCA	HTX	Laterality defect (*N*)	11	0.0132	0.4074	6.9342	0.0324	6.9342	1.5884	5.9480 × 10^−8^
CHD_NCA	Laterality defect (*N*)	HTX	11	0.0132	0.2245	6.9342	0.0588	6.9342	1.2477	5.9480 × 10^−8^
CHD_NCA	Vasc ring	Respiratory (*N*)	13	0.0156	0.3421	5.1876	0.0456	5.1876	1.4198	1.6641 × 10^−7^
CHD_NCA	Respiratory (*N*)	Vasc ring	13	0.0156	0.2364	5.1876	0.0659	5.1876	1.2499	1.6641 × 10^−7^
CHD_NCA	RVOTO, laterality defect (*N*)	CTD	9	0.0108	0.6923	4.8115	0.0156	4.8115	2.7824	8.6630 × 10^−6^
CHD_NCA	CTD, laterality defect (*N*)	RVOTO	9	0.0108	0.4286	4.1084	0.0252	4.1084	1.5674	1.0061 × 10^−4^
CHD_NCA	VSD, PDA, CNS (*N*)	LVOTO	10	0.0120	0.2326	3.6595	0.0516	3.6595	1.2202	1.7389 × 10^−4^
CHD_NCA	VSD, LVOTO, CNS (*N*)	PDA	10	0.0120	0.8333	3.4236	0.0144	3.4236	4.5396	2.5161 × 10^−5^

^a^ Association analysis performed with the apriori principle to find rules; rules with a lift >3 were considered valuable.

^b^ Association analyses for CHDs and NCAs involved 887 subjects with associated CHDs.

^c^ lhs, left-hand side; rhs, right-hand side. A rule can be written as lhsrhs, “lhs” and “rhs” are collections of phenotypes that lie at the end of the arrow.

^d^ Support = count (number of specific rule)/total subjects observed, represents rule frequency, and the numerator of the formula can be “lhs” or “rhs” as well; confidence = support(lhs∪rhs)/support(lhs), represents the probability “rhs” coexistence in subjects with “lhs”; lift = support(lhs∪rhs)/(support(lhs)*support(rhs)), measure of independence of “lhs” and “rhs”.

^e^ Quality measurement: Fisher's Exact Test inspects rule authenticity (rule valid at *P* < 0.05).

^f^ When inspecting “CHD-NCA” rules, phenotypes with “(*N*)” refer to NCAs, and others represent CHDs.

## 4. Discussion

In the present study sample, 23.42% of CHD diagnosed children had coexisting NCAs, a value about midway between the wide range of percentages reported in the literature (4.53%–50%) (13–15) and somewhat similar to percentages obtained in extensive studies conducted in the city of Atlanta, GA in the USA (28.7%), the province of Alberta in Canada (25%), and Croatia (14.5%) (16, 26, 27). The wide range of findings reported in this regard may be attributed to different classifications, inclusion/exclusion criteria, and screening strategies. Stoll and colleagues reported that the incidence of NCAs co-occurring with CHDs was 24.2% in live births, 66.3% in stillbirths, and 69.4% in abnormal pregnancy terminations (21). The subjects in our study were all live births. The definition of associated CHD differs between studies as well. Some defined an associated CHD as an extracardiac or genetic abnormality (16, 21). In our study, we included anomalies affecting other organs but not genetic abnormalities. Consequently, the data are not fully comparable across different studies due to different methodologies. Nevertheless, the proportion of congenital defects was dramatically more significant than the prevalence of congenital defects for China's population as a whole (191.84/10,000) (28), which suggests that CHDs may be a risk factor for other defects and that a considerable number of children with CHDs have NCAs.

Our findings indicating that the most common NCAs observed were CNS anomalies, nose/ear/mandibular/face anomalies, genitourinary system anomalies, and musculoskeletal system anomalies are largely in agreement with previous reports (14, 21, 29–31). However, we observed relatively low percentages of gastrointestinal anomalies (6.31%) and respiratory system anomalies (6.20%), the ranges for which in the literature are 8%–35% and 2%–14%, respectively, but a relatively high percentage of nose/ear/mandibular/face anomalies (19.39%) (8, 14, 15, 18, 32–39). Divergences from prior reports may have several underlying causes. Firstly, we excluded stillbirths, fetal deaths, and pregnancy terminations, which are more likely to involve lethal and major anomalies than live births. Secondly, we included several minor NCAs that are often excluded in other literature (e.g., low set ears, atypical facial appearance, and high arched palate) because how little of an effect they have on prognoses. Lastly, we deconstructed phenotypes of known syndromes as discrete NCAs. Most syndromes that commonly present with CHDs—including Down syndrome, DiGeorge syndrome, and Turner syndrome—have clinically significant NCAs, such as development delays, atypical facial features, skeletal deformities, and genitourinary system anomalies (40); these syndromes explain in part the top four NCAs in our results. CNS anomalies, including development delays, account for a large portion of the NCAs observed in our study (Table 2). Because neurodevelopment is highly sensitive to oxygen, any cardiac defect that disturbs hemodynamics and oxygen transport has the potential to cause CNS anomalies. In addition, development delays continue to manifest with increasing child age; our study sample has a median age of 3.96 years (IQR, 2.56–5.39), providing recognition of NCAs with progressive symptoms that are not yet apparent in the fetal and infant stages. Furthermore, adverse neurodevelopmental outcomes after surgical repair of CHDs represent a clinically significant cause of morbidity. Indeed, neuropsychological deficits may occur in as many as 50% of children who undergo CHD repairs by the time they reach school age (41). Early diagnosis of CNS anomalies in this patient population would be of great benefit to perioperative management.

Regarding NCA risk among different CHD phenotypes, we found that AVSD, PTA, vasc ring malformations, LVOTO, and heterotaxy had relatively high incidences of associated NCAs, whereas VSD and APVD had relatively low incidences. It could be that CHDs that emerge in earlier stages of morphogenesis are more likely to be associated with multiple and complex defects (42). Boundaries between tissues are blurred in early development, and regulatory molecular signaling pathways affect the development of many organs simultaneously.

Our finding of a specific link between VSD/LVOTO/PDA presence and CNS anomalies should be explored. Interestingly, the impact of AVSDs on CNS anomalies and nose/ear/mandibular/face anomalies could potentially relate to the well-known correlation between AVSDs and Down syndrome (32, 43, 44). The finding that vasc ring anomalies (including right arch and sling) often co-occurred with malformations of the respiratory system was unsurprising given the oppression of the malformed arteries on the trachea. Theoretically, most CHD-associated NCAs should have an etiological explanation, such as a mesoderm differentiation event that causes CHD and musculoskeletal defects simultaneously (16). The detailed mechanisms underlying the breadth of co-occurring anomalies have yet to be delineated.

## 5. Limitation

It was difficult to conduct a stratified analysis to correct for confounding factors as we did not obtain data related to risk factors for birth defects. We also did not capture associated CHD patterns in rural China, where there is a high prevalence of unrecognized CHDs (32). Thirdly, our sample included relatively few rare CHDs and NCAs, which may weaken the analyses.

## 6. Conclusion

Han Chinese children with CHDs were found to have a high prevalence of NCAs, including CNS, nose/ear/mandibular/face, and musculoskeletal anomalies. Different CHD subtypes had different NCA probabilities and spectrums. Compared to other CHD subtypes, septal defects had a lower percentage of associated NCAs while AVSD had a higher percentage. Clinicians treating patients with CHDs should be attentive to the risk of NCAs, particularly for perioperative management but also for long-term prognosis determination.

## Data Availability

The original contributions presented in the study are included in the article/Supplementary Materials, further inquiries can be directed to the corresponding authors.
